# Dietary Niche Variation in an Invasive Omnivore: The Effects of Habitat on Feral Pig Resource Use in Hawai‘i

**DOI:** 10.1002/ece3.70417

**Published:** 2024-10-16

**Authors:** Michael S. Peyton, Kealohanuiopuna M. Kinney, Sarah Knox, Grace Tredennick, Sara Hotchkiss

**Affiliations:** ^1^ University of Wisconsin‐Madison, Botany Madison Wisconsin USA; ^2^ Institute for Pacific Islands Forestry United States Forest Service Hilo Hawaii USA

**Keywords:** diet, dietary niche, feral pig, Hawaii, omnivore, specialization, stable isotope, *Sus scrofa*

## Abstract

Invasive omnivores may have profound impacts on ecological communities through diet selection, particularly when their functional roles differ from those in their native range. While the threat of feral pigs (*Sus scrofa*) to native plant communities in Hawai‘i is well known, their trophic dynamics and the drivers of variation in their diet remain understudied. We investigated the feral pig trophic niche on Hawai‘i Island using stable isotopes (^13^C and ^15^N) and Bayesian mixing models to identify drivers of variation in resource use. We also reconstructed intra‐individual variability for six subsampled individuals to understand temporal variation in resource use and individual diet specialization. Our results revealed that feral pigs on Hawai‘i Island exhibit a broad trophic niche characterized by diverse diets, with substantial overlap in resource use across districts and habitats. Differences in dietary composition in the transition from forest to open habitat were driven primarily by a decline in invertebrates and an increasing reliance on resources enriched in ^15^N, which may reflect a shift in protein sources with habitat. Pigs in forested areas exhibited a smaller trophic niche than those in open habitats, largely driven by differences in feeding strategies and resource availability. Diets for subsampled individuals varied little, suggesting feral pig resource‐use strategies in Hawai‘i tend to be relatively stable through time. Individual niche width was relatively narrow compared to that of feral pigs in Hawai‘i at large, indicating the relatively wide feral pig dietary niche is characterized by substantial intraspecific diet specialization, likely as a result of strong intraspecific competition. Understanding the drivers of feral pig resource use offers key information for management strategies aimed at mitigating their ecological impacts in imperiled systems like Hawai‘i.

## Graphical abstract

We investigated the feral pig dietary niche on Hawai‘i Island using stable isotopes (^13^C and ^15^N) and Bayesian mixing models to identify drivers of variation in resource use. Primary differences in resource use were between pigs in forest and open habitat, largely driven by ^15^N enrichment in open habitat, likely as a consequence of shifting protein sources. We found evidence for substantial individual diet specialization, likely as a result of strong intraspecific competition from high population sizes.

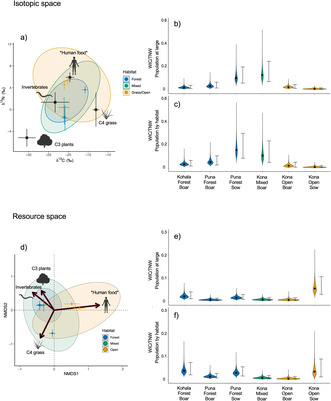

## 1 Introduction

Despite the role of omnivores in shaping ecosystems worldwide, their trophic dynamics are relatively understudied (Hamalainen et al. 2022). Omnivory is generally associated with a broad dietary niche (Rozin 1976), but variation in occupied niche space can be considerable, even within a species (Layman and Allgeier 2012; Newsome et al. 2009; Paolini et al. 2018). Resource quality and availability (Hamalainen et al. 2022; Manlick and Pauli 2020), intraspecific and interspecific competition (Galetti et al. 2015; Milinski 1982; Morse 1974), and individual past experience (Garcia et al. 1974; Rogers and Blundell 1991; Rozin 1976) can all significantly influence a consumer's dietary niche by affecting resource selection and subsequently altering trophic interactions. Even when omnivores and other generalist consumers occupy a broad niche at the population level, how niche space is partitioned among individuals can differ substantially (Bolnick et al. 2003). Recent work has highlighted the importance of variation within and across individuals to understand the dietary ecology of generalist consumers (e.g., Larson et al. 2020; McEachern et al. 2006; Scholz et al. 2020), and it is becoming increasingly evident that effects driven by intraspecific variation in resource use can propagate through food webs to fundamentally influence community structure (Newsome et al. 2009; Scholz et al. 2020; Svanbäck and Persson 2004) and function (Benkendorf and Whiteman 2021; Křivan and Diehl 2005). Filling these gaps is especially important in the case of invasive omnivores in ecosystems where their functional roles are poorly understood.

Feral pigs (*Sus scrofa*) are among the most destructive and widely introduced invasive species globally (McClure et al. 2018) and have dramatically altered ecosystems worldwide (Wehr et al. 2018). As omnivores and diet generalists, feral pigs exploit a large variety of resources, which not only supports their establishment and spread in a wide range of climates and habitats (Ballari and Barrios‐García 2014), but also allows pigs to shift their diets temporally based on seasonal resource availability (Loggins et al. 2002; Wilcox and Van Vuren 2009; Wurster et al. 2012). Seasonal variation in pig diet can lead to a dynamic restructuring of trophic impacts through time (McMeans et al. 2019). For example, feral pigs have directly led to widespread declines and, in some cases, extinction of native plant and animal species due to herbivory (Murphy et al. 2014), predation (Whytlaw, Edwards, and Congdon 2013), and competition (Galetti et al. 2015), and have indirectly contributed to the spread of other non‐native species through biotic disturbance and dispersal (Peyton, Rodriguez Curras, and Hotchkiss 2023). When available, human food subsidies constitute a significant portion of the feral pig diet, often resulting in the destruction of agricultural and residential property (Herrero et al. 2004).

In Hawai‘i, like other island systems with no history of large mammalian herbivores, feral pigs are largely unconstrained by competition and predation. Under these conditions, pigs have created novel top‐down forcing for native communities with naïve species lacking adaptations to such conditions (Cordell et al. 2009; Scheffler et al. 2012), which has resulted in significant changes to species composition in forests where they encroach (Cole et al. 2012; Murphy et al. 2014). Weaker interspecific competition for pigs in these systems may promote higher inter‐individual variation and the expansion of their trophic niche (Bolnick et al. 2003; Van Valen 1965). This variation and diet expansion often result in trophic impacts distributed heterogeneously across individuals and populations (Bolnick et al. 2010; Lunghi et al. 2020).

While substantial effort has focused on the dramatic effects of pigs on Hawaiian flora and fauna, there are relatively few investigations on the feeding ecology of pigs or the drivers of variation in diet. Exploring pig trophic dynamics is especially important on an ecologically diverse island like Hawai‘i Island (the “Big Island”), where steep gradients in climate and vegetation generate significant variation in habitat type (Mueller‐Dombois 1988). Feral pigs have invaded the majority of Hawai‘i Island, and the ecological diversity of the island has likely led to differences in resource use and home‐range size among populations (Diong 1982). Generalists can persist in otherwise unsuitable habitats with sufficient subsidies from anthropogenic resources (Larson et al. 2020; Pedrosa et al. 2021), which may allow them to overcome constraints associated with macronutrient availability (Cervo and Guadagnin 2020; Stillfried et al. 2017). Utilization of human food resources is commonly documented (Wehr et al. 2018) and is likely driven by risk‐reward trade‐offs (Houston, McNamara, and Hutchinson 1993) and the availability of other resources. Seasonality may also influence resource availability and dietary niche breadth; in Hawai‘i, seasonality is characterized by wet (November through April) and dry (May through October) periods, with the greatest seasonal moisture variation in areas where mean annual precipitation is low (Giambelluca et al. 2013). Indicators of spatial and temporal patterns in resource use can provide valuable context to identify the functional role that feral pigs occupy under conditions far removed from their native range.

Here we quantify the feral pig trophic niche on Hawai‘i Island using stable isotopes of carbon and nitrogen from tail hairs collected by hunters across the island. Stable isotope analysis has led to significant advances in dietary ecology in recent years (Bicknell et al. 2020; El‐Sabaawi et al. 2009; Happel et al. 2015; Layman and Post 2008; Shiels et al. 2013; Vaudo and Heithaus 2011). Stable isotopes allow us to quantify dietary composition via the isotopic distinction of resources in a consumer's diet that have become assimilated into inert tissues (Moore and Semmens 2008; Newsome et al. 2007; Phillips and Gregg 2003). Ratios of stable carbon, reflecting ^13^C discrimination based on biochemical differences in carbon fixation during C3 and C4 photosynthesis, and stable nitrogen isotopes, tracking trophic position with ^15^N enrichment at higher trophic levels, are especially useful in dietary analysis due to their direct links to trophic processes and their high abundance in biological tissues (Peterson and Fry 1987). We estimated the dietary composition of individuals captured in different districts and habitats using Bayesian mixing models and identified factors influencing resource use. Furthermore, we reconstructed intra‐individual resource‐use patterns for a subset of six sequentially subsampled individuals to quantify resource niche heterogeneity and individual diet specialization. We predicted that isotopic signatures and dietary composition differ across habitats and districts due to differences in resource availability. Furthermore, we expected that the feral pig dietary niche space in drier areas with open vegetation would be larger than that of wetter districts and forested habitats due to greater spatial and temporal heterogeneity in the availability of resources in drier areas.

## 2 Methods

### 2.1 Study System

**FIGURE 1:**
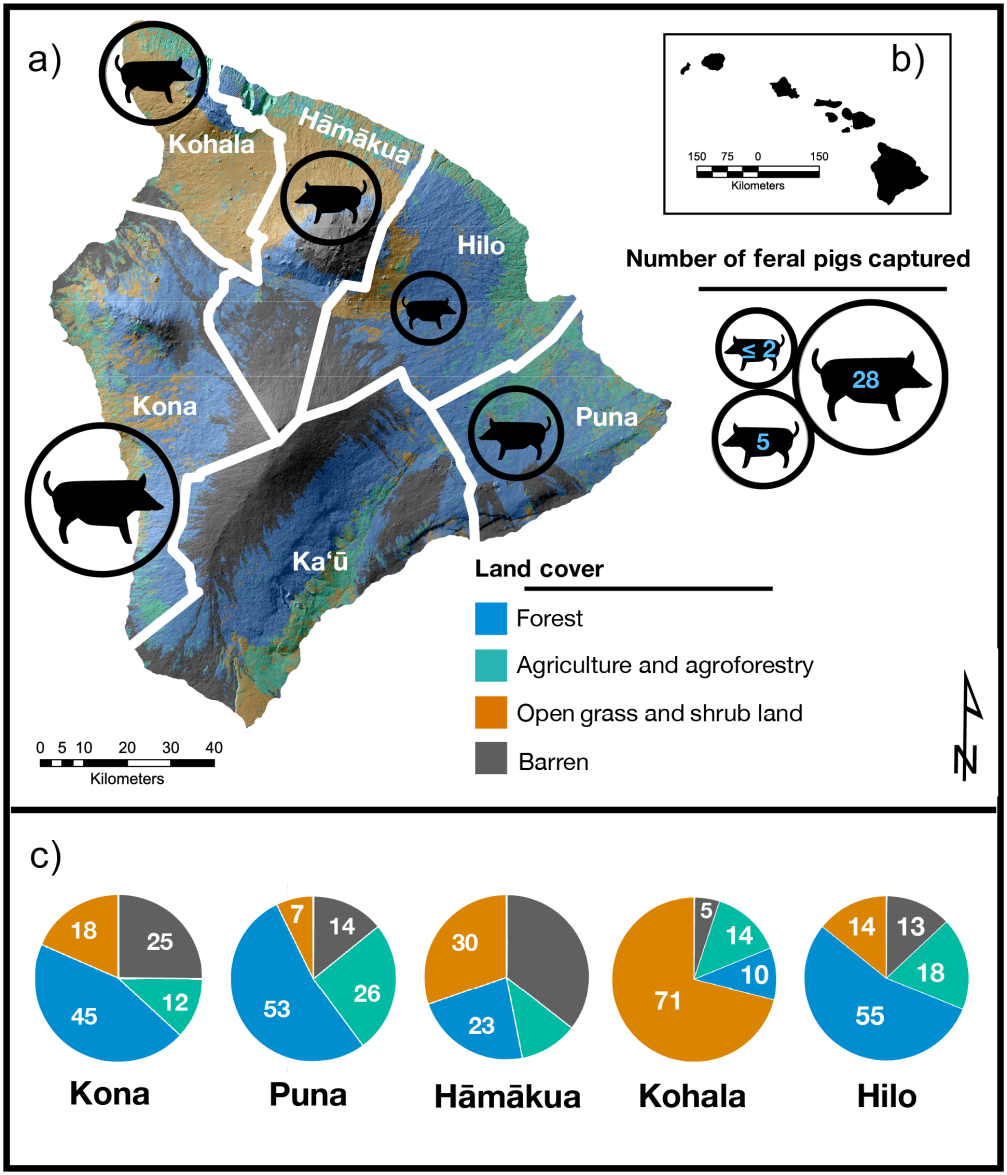
Study area: (a) Map of captures by district, (b) inset of Hawaiian islands, (c) relative percentage of vegetation in each district (Hawai‘i Land Cover and Habitat Status CAH 2017). No pigs were captured in Kaʻū.

We obtained pig hair samples from hunters across the island in partnership with the United States Forest Service (USFS) and the Keiki of Da ʻĀina nonprofit organization on Hawai‘i Island. Feral pigs were captured from 5 of 6 districts representing *moku*, or traditional Hawaiian land divisions (Puna, Hāmākua, Kohala, Kona, and Hilo; no pigs were captured in Kaʻū district). Each district aggregates adjacent watersheds, spans a wide elevational gradient, and contains considerable heterogeneity in land cover (Figure 1). The Kona, Puna, and Hilo districts are characterized by approximately 50% forest cover, whereas Hāmākua and Kohala have notably less (23% and 10%, respectively; Hawai‘i Land Cover and Habitat Status CAH 2017). The Kohala district features a substantially greater percentage of open habitat than other districts due to the presence of pasture and ranchland, along with converted forest and shrubland, both of which are largely dominated by non‐native C4 grasses (Chadwick et al. 2007). Agriculture and agroforestry practices are present to varying degrees in each district but cover the greatest area in Puna (> ¼ of land cover). Dramatic agricultural shifts in the past 40 years have led to significant declines in sugar and pineapple production, with a concurrent rise in coffee, macadamia nuts, and local crop cultivation (taro, tropical fruits, etc.), along with the expansion of commercial eucalyptus plantations in former sugarcane fields along the northeast coast in the Hāmākua and Hilo districts (Perroy, Melrose, and Cares 2016). Hilo and Kona are the most populous districts as of 2020, with populations of roughly 53,600 and 53,100, respectively (US Census Bureau 2023). These human populations are largely centered around the city and town of Hilo and Kailua‐Kona, respectively. Pigs are found in all districts and occupy a variety of habitats. These include low‐elevation mesic and wet forests dominated by non‐native C3 vegetation, high‐elevation montane forests with varying degrees of intact native C3 vegetation, and open shrub and grassland with abundant non‐native C4 grasses (Barton et al. 2021).

### 2.2 Sample Collection and Preparation

Feral pig hair samples were collected on April 1^st^ 2023 during the 10^th^ annual Keiki of Da ʻĀina pig hunting tournament, in which teams from across the island tracked and captured pigs within a 12‐h window. We collected samples from the tail hair of 48 feral pigs, and information on sex, age (by dentition), weight, and body length were recorded. We distinguished between castrated males (Laho‘ole; castrated and released as piglets) and non‐castrated males. Due to time constraints, we were unable to collect age and size data for all individuals. Hunters provided information about the district where pigs were collected, as well as the habitat where they were found (forest, open, and mixed—i.e., intermediate habitats between forest and open). Only 2 individuals were captured in Hilo; thus, they were removed from analyses among districts. While the spatial resolution and habitat information were coarse and we cannot guarantee they reflect the long‐term conditions experienced by each individual, recent resource use likely reflects local conditions. Home‐range estimates for pigs vary widely based on habitat and resource availability, ranging from 0.62 to 48.3 km^2^ globally (Garza et al. 2018). Direct estimates from radio‐collared individuals in Hawai‘i are smaller than average global estimates, ranging from 0.62 to 2.01 km^2^ under forested conditions (Anderson and Stone 1993; Diong 1982) and from 5.18 to 10.36 km^2^ in open habitats (Griffin 1978). Maximum home‐range estimates do not exceed the minimum area encompassed by districts (1255 km^2^ for Kohala; Figure 1), though habitat type varies within districts. Although more recent direct estimates are, to our knowledge, lacking in Hawai‘i, Risch, Honarvar, and Price (2022) found significant seasonal shifts in abundance across ecotones in Maui, suggesting pigs may migrate longer distances seasonally due to changes in resource availability and hunting pressure. Nevertheless, it is reasonable to assume recent (i.e., 5–7 days) sources of C and N assimilated into inert tissues were derived from resources near the capture location for most individuals (Wurster et al. 2012).

Pigs consume a wide range of food resources, many of which cannot be distinguished isotopically. To determine the isotopic signatures of potential resources, we opportunistically collected samples of common resource categories known to be consumed by feral pigs on Hawai‘i Island, including native C3 plant tissues (fruits, leaves, roots; *n* = 20), non‐native C4 pasture grasses (leaves, roots; *n* = 12), and invertebrates (i.e., earthworms and other detritivores; *n* = 7) from across the island (Table S1), while recognizing that some native C4 plants and non‐native C3 plants may contribute to pig diet. We ran separate MANOVAs for C3 and C4 plants to identify differences in isotopic signatures among tissue types (i.e., leaves, roots, and fruits), and tissues did not differ significantly within either C3 or C4 plants. We proceeded using C3 plants, C4 plants, and invertebrates as resource categories in our analyses. Samples were collected primarily in the Kohala and Hilo districts, which differ considerably in climate and substrate age (Giambelluca et al. 2013). However, MANOVA showed that isotopic signatures did not vary significantly between sampling locations within resource categories (Table S2), indicating differences due to climate and substrate age are far exceeded by those due to resource category. To include estimates for possible anthropogenic resource consumption, we used values of δ^13^C and δ^15^N for United States residents from Hülsemann et al. (2015) corrected for trophic enrichment. Feral pigs encounter a wide range of anthropogenic resources in the form of food refuse, agricultural crops, and intentional supplementation by hunters to promote growth (Ballari and Barrios‐García 2014). While using corrected human isotopic values does not permit discrimination among these specific categories, it does provide a method of incorporating broad estimates of anthropogenic inputs. While human consumption of marine resources is likely higher in Hawai‘i than in the continental United States, using values from Asian countries with higher fish consumption—and thus slightly higher δ^15^N and lower δ^13^C values—provided no qualitative differences in our results. In addition to anthropogenic resources, feral pigs are known to consume a wide range of other resources typically found at higher trophic positions (Wehr et al. 2018) and with a similarly elevated δ^15^N signature. These include ground‐nesting seabirds, eggs (Nogueira‐Filho, Nogueira, and Fragoso 2009), and scavenged interspecific and intraspecific animal material (Cukor et al. 2020). We note here that consumption of these resources contributes largely to estimates of human food resource use in our models, and thus should be considered as a possible contributor to this category.

Pig hair samples were cleaned using a 2:1 chloroform: methanol solution, sectioned, and ^13^C and ^15^N were quantified using accelerator mass spectrometry at the University of New Mexico's Stable Isotope Laboratory (Albuquerque, NM). We used 3‐mm sections of the most recently grown hair (i.e., closest to the skin) for analyses exploring how resource use varied with spatial location. We also sequentially sampled hair from six individuals representative of a range of conditions (i.e., sex, capture district, and habitat type) to explore temporal variation in dietary composition within individuals. 3‐mm segments were taken at each 9‐mm interval except for one individual (i.e., the boar captured in the Puna district), where the last four segments were taken contiguously to sufficiently sample across a shorter total length. 12–16 subsamples were taken from each individual for a total of 85 subsamples. As all individuals were captured and harvested on the same day, we assumed 3‐mm sections of hair represented assimilation across a similar interval among individuals, with the understanding that hair growth rates may vary.

### 2.3 Statistical Analyses

#### 2.3.1 Spatial Variation

Bayesian versions of mixing models provide a method of estimating the contributions of resources to a consumer's diet while incorporating the inherent variability in isotopic values, C and N concentration, and trophic discrimination factors (Parnell et al. 2013). We applied separate Bayesian mixing models to samples of recently grown hair for each individual using the *simmr* package (Goven and Parnell 2018) in R (R Core Team 2023), using trophic discrimination factors (TDFs) of −2.3 ± 0.5 and 3.5 ± 0.5 (mean ± sd) for δ^13^C and δ^15^N, respectively. The models were run using three chains with 100,000 iterations with 20,000 removed for burn‐in and sample thinning by a factor of 100 to reduce autocorrelation. One individual with an abnormally high δ^15^N value (12.9 before applying TDFs) was outside of the mixing space and was removed from the analysis. Median resource contribution values from the resulting posterior distributions were extracted to estimate the average dietary composition for each individual.

As resource availability and behavioral differences influence variation among individuals in dietary composition (Rogers and Blundell 1991), we tested for differences in both isotopic signatures and estimated dietary composition among sexes, capture districts, and capture habitats. To do this, we used PERMANOVA with pairwise comparisons using Bonferroni correction in the *vegan* (Philip 2003) and *pairwiseAdonis* packages in R (Martinez Aribizu 2020). Age, weight, and body length were not sufficiently sampled to appropriately analyze differences across age and size classes. Among groups identified as dissimilar, we used Similarity Percentage (SIMPER) analysis from the *simper* function in the *vegan* package to identify which resources primarily contributed to those differences. Resources driving variation between groups play a disproportionate role in determining differences in diet preferences and trophic impacts across populations. To characterize the dietary niche in resource space, we ordinated resource estimates for individuals using non‐metric multidimensional scaling (NMDS) with Bray–Curtis distance. Next, we quantified dietary niche width using estimated Bayesian standard ellipse areas (SEA; a measure of niche breadth) corrected for small sample size (SEAc) using the R package *SIBER* (Jackson et al. 2011) and calculated niche width among sexes, capture districts, and capture habitats in both isotopic (i.e., δ^13^C and δ^15^N) and resource (i.e., NMDS) space. Models were run using three chains for 100,000 iterations, with 2000 removed for burn‐in and thinning by a factor of 100.

#### 2.3.2 Individual Diet Specialization

To understand diet flexibility within individuals, we ran Bayesian mixing models on the six sequentially subsampled individuals, with individual models for each 3‐mm hair segment. Hair growth rates are, to our knowledge, unknown for pigs in Hawai‘i, and very few estimates have been calculated for feral pigs in general. Applying a hair growth rate estimate of 0.4 mm day^−1^ from Wurster et al. (2012) and Cerling and Viehl (2004), each 3‐mm segment represents 7.5 days. While this growth rate estimate is not sufficiently calibrated for our study site to reconstruct precise temporal trends, we can reasonably assume each segment represents a relatively short (~1 week) period of assimilation, and comparisons of same‐length segments at different positions can be used to quantify temporal diet variability within individuals.

To quantify the degree of individual diet specialization, we estimated niche breadth for the six subsampled individuals in relation to total, population‐wide niche breadth in both isotopic and resource space following Bolnick et al. (2002). In this formulation, individual diet specialization can be quantified by the ratio of the within‐individual component (WIC) of niche width to the total niche width (TNW) of the population, expressed as WIC/TNW. Individuals with low WIC/TNW occupy a small portion of niche space in relation to the total niche breadth. It is important to recognize that this measurement of diet specialization, sensu Bolnick et al. 2002, differs from the designation of diet specialists vs. diet generalists related to the evenness in the distribution of resource contributions, sensu Newsome et al. (2012), and provides an index of individual diet variability relative to the population. We calculated WIC/TNW from Bayesian standardized ellipse areas corrected for small sample size (SEAc's) in both isotopic and resource space using the R package *SIBER* (Jackson et al. 2011). Models were run using similar parameters as those above to quantify SEAc's for populations delineated by district and habitat. We compared SEAc's for each of the six subsampled individuals to (i) the total feral pig dietary niche, (ii) the dietary niche of pigs found in the same district, and (iii) the dietary niche of pigs found in the same habitat.

## 3 Results

### 3.1 Spatial Variation

**FIGURE 2:**
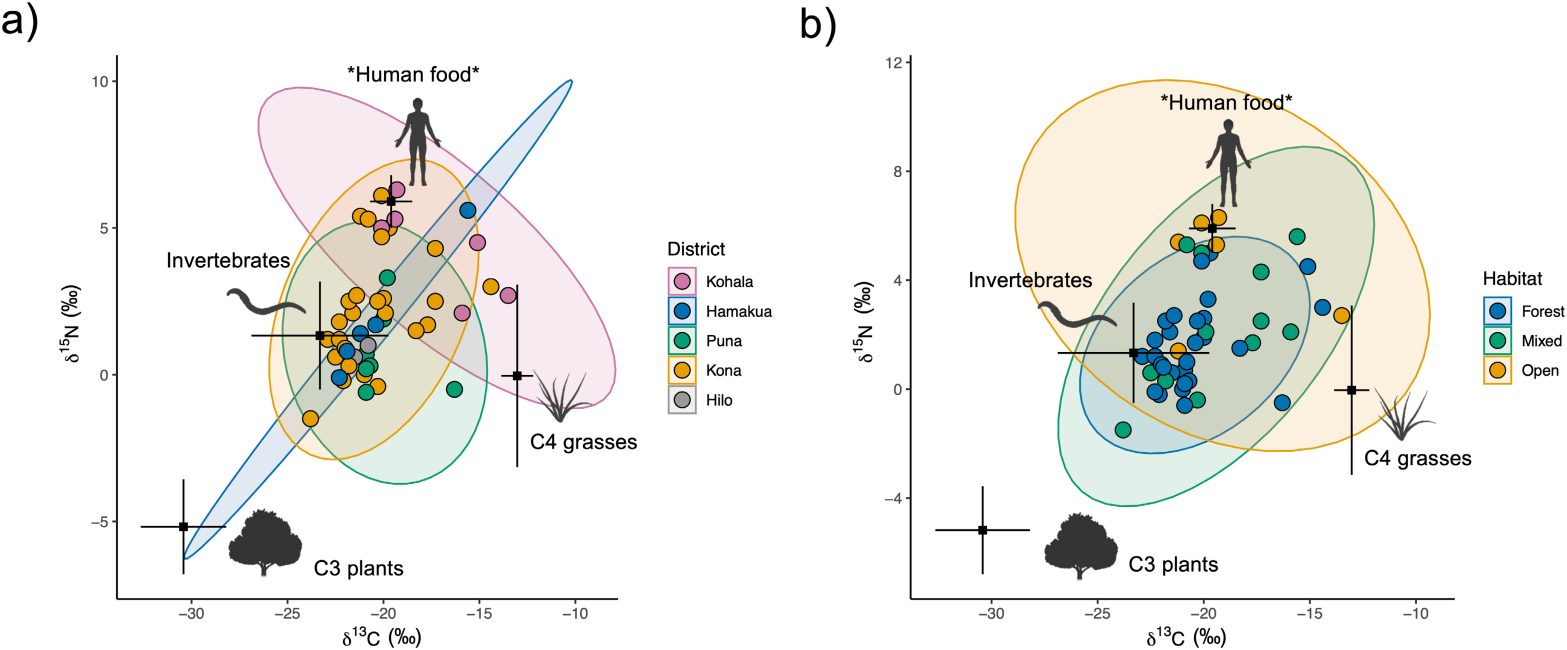
Isotope plots for raw δ^13^C and δ^15^N values of sampled individuals. Ellipses represent 95% confidence intervals for populations delineated by (a) capture district and (b) capture habitat. Black dots show the means and 95% confidence intervals for resource categories (C3 plants, C4 grasses, invertebrates, *human food*) after applying trophic discrimination factors. *Human food* may also represent the inclusion of other resources found at higher trophic positions.

Individual pigs varied considerably in isotopic space, ranging from −26.1 to −15.8‰ and 2.0 to 9.8‰ for δ^13^C and δ^15^N, respectively (Figure 2). We observed substantial overlap among pigs sampled in different districts and habitats, each exhibiting a broad range of isotopic values. Pigs found in the Kohala district and open habitats appeared to exhibit higher δ^13^C and δ^15^N values than in other districts and habitats, potentially indicating a higher reliance on C4 grasses and resources of higher trophic position such as scavenged animal material or human food subsidies. PERMANOVA showed separation across both district and habitat distinctions in isotopic space, but no differences among sexes (Table 1). Pigs from Kohala differed in their isotopic composition from those in Kona and Puna, while pigs found in forests differed from those in open habitats. SEAc values across habitat distinctions showed that pigs in forests occupied the smallest isotopic niche width, followed by those in mixed habitats, and those in open habitats occupied the broadest isotopic space (Table S3).

**TABLE 1:** Results of PERMANOVA with pairwise comparisons between districts, habitats, and sexes (*laho'ole* signifies castrated males).

	Isotopic space		Resource space	
	*R* ^2^	*p* (adjusted)	*R* ^2^	*p* (adjusted)
District				
Kohala vs. Kona	**0.226**	**0.005**	**0.153**	**0.026**
Kohala vs. Puna	**0.451**	**0.005**	0.311*	0.088*
Kohala vs. Hāmākua	0.297	0.358	0.262	0.406
Kona vs. Puna	0.048	0.905	0.042	1.000
Kona vs. Hāmākua	0.002	1.000	0.023	1.000
Puna vs. Hāmākua	0.050	1.000	0.015	1.000
Habitat				
Forest vs. Open	**0.176**	**0.013**	**0.220**	**0.003**
Mixed vs. Open	0.093	0.609	0.137	0.308
Forest vs. Mixed	0.043	0.538	0.046	0.450
Sex				
Male vs. Female	0.012	1.000	0.007	1.000
Female vs. Laho'ole	0.014	1.000	0.019	1.000
Laho'ole vs. Male	0.003	1.000	0.009	1.000

*Note:* Bolded values are significant at *p* < 0.05 with *p* values adjusted using Bonferroni correction, bolded values marked with an asterisk are weakly significant at *p* < 0.10.

**FIGURE 3:**
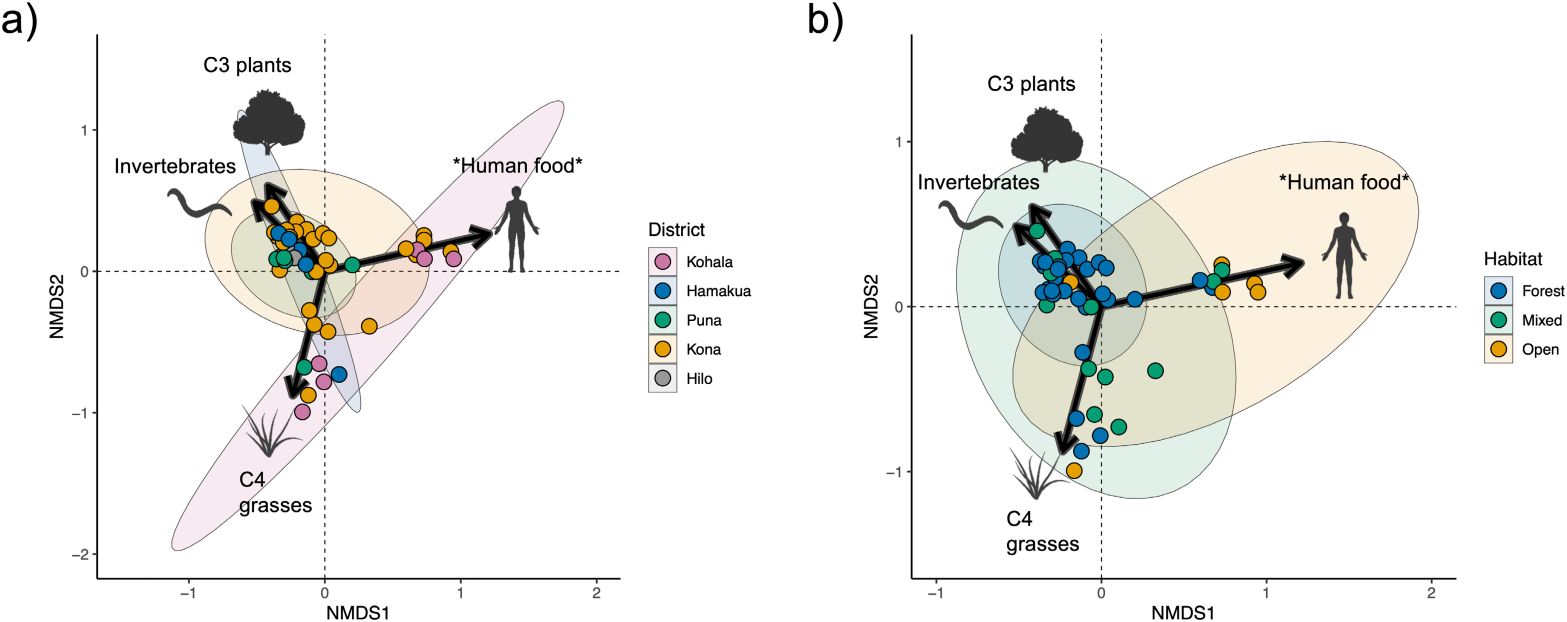
Results of NMDS quantifying relationships among individuals in resource‐space using Bray–Curtis distance. Vectors signify resources, with lengths scaled to vector loadings on NMDS1 and NMDS2. Ellipses represent 95% confidence intervals for populations delineated by (a) capture district and (b) capture habitat.

Mixing models revealed high variability in estimated resource consumption patterns, supporting our expectation of diverse diets and a broad niche for feral pigs across Hawai‘i Island (Figure 2). Similarly to metrics derived from raw isotopic values above, SEAc values from resource estimates increased across habitat distinctions from forest to open habitat (Table S3). Estimates from mixing models and PERMANOVA showed somewhat similar patterns to those in isotopic space across capture districts and habitats (Table 1). Differences were again found between pigs in open and forested habitats, but only pigs in the Kohala and Kona districts were found to differ significantly, with weakly significant differences between Kohala and Puna (Table 1). SIMPER showed all resources contributed to differences in dietary composition between pigs found in the Kohala and Kona districts (Table S4). Human foods (hereafter designated with asterisks when highlighting the possible inclusion of other resources enriched in ^15^N, that is *human foods*; 0.18 ± 0.11, *p* < 0.001) and invertebrates (0.09 ± 0.05, *p* < 0.001) primarily drove differences in resource use in open and forested habitats (Figure 3; Table S4). Furthermore, NMDS suggests resource use for pigs in Kohala and open habitats tends toward higher values of *human food* than in other districts and habitats (Figure 2c,e), although findings may be confounded by differences in sample sizes.

### 3.2 Individual Diet Specialization

Sequentially sampled individuals demonstrated minor changes in isotopic values and resource use estimates over time (Figure 5). We observed qualitative differences in dietary composition among districts and habitats that mirrored results at the population level, except for the individuals captured in a forested habitat in Kohala. The two individuals captured in the Puna forest habitat and the individual captured in the South Kona mixed habitat exhibited isotopic values and resource use estimates similar to others captured in the forest and mixed habitats, respectively. The two individuals found in the Kona open habitat expressed elevated δ^13^C and δ^15^N values, with mixing models estimating large contributions from possible animal tissues and human food, contrasted by relatively low proportions of other resources. The individuals captured in the Kohala forest exhibited patterns indicating a high reliance on C4 grasses, differing from most individuals captured in forest habitat but resembling others captured in the Kohala district.

The dietary niche width of individuals—measured by both raw isotopic values and by resource estimates—was small compared to that of the total feral pig niche width and that of all pigs captured in the same district or habitat (Figure 5, Table S5). WIC/TNW values were slightly larger on average in isotopic space than in resource space, and while minor differences were present among individuals in the two metrics, the overall trend indicated high individual specialization. Median WIC/TNW estimates ranged from 0.01 to 0.13 in isotopic space and 0.01 to 0.06 in resource space when TNW was estimated from the total population niche width, and values were similar when TNW was estimated as that of the capture district or habitat (Table S5), although with some rank‐reversals.

## 4 Discussion

Feral pigs are notorious for their diet flexibility, which contributes to their invasion of a wide range of habitats globally (Barrios‐Garcia and Ballari 2012). Our findings show populations in Hawai‘i are no exception, with feral pigs collectively inhabiting a broad dietary niche (Figure 2). Notably, δ^15^N values indicated these pigs occupy a wide range of different trophic positions, with some individuals exhibiting values typically expressed by terrestrial scavengers and carnivores (Rodriguez Curras et al. 2022). High values are likely driven by a reliance on animal tissues and human food subsidies with δ^15^N values exceeding those typically found in other commonly utilized resources (Hülsemann et al. 2009). Resource estimates from mixing models support these initial observations, with feral pigs exhibiting a wide range of resource combinations and demonstrating several distinct foraging strategies across populations. We recognize that these estimates are subject to varying degrees of uncertainty, and we acknowledge that estimate precision for C3 plants and invertebrates is limited for some individuals due to their position in isotope space relative to the configuration of these resources (Newsome et al. 2012). Given that pigs typically prefer vegetative material over animal material (Senior et al. 2016), model underestimation of C3 consumption may explain why our results show a lower reliance on C3 plant material than we expected in forest habitats. However, studies show that compared to their native range, animal material constitutes a larger portion of the feral pig diet in their introduced range (Ballari and Barrios‐García 2014), and individual diets can vary widely. Nevertheless, both model estimates and raw isotopic values support a wide range of resource‐use patterns and demonstrate the considerable intraspecific variability in diet selection among pigs across Hawai‘i Island.

### 4.1 Spatial Variation in Resource Use

We found substantial overlap in resource use across capture districts and habitats (Figure 2). This is certainly due, in part, to our coarse spatial resolution for capture location, which is intentionally imprecise so hunters can protect their choice hunting grounds. Our spatial resolution is therefore limited to districts, which are often highly heterogeneous and include multiple habitat types. In addition, accuracy in habitat designation was difficult to determine even with relatively broad classifications since hunters may lack a shared definition of habitat distinctions. We nevertheless observed distinct patterns in resource use among pig populations separated by district and habitat, indicating site use and diets were sufficiently spatially constrained that distinctions can be detected even with such coarse designations. We observed that habitat classification was particularly informative in shaping resource use, suggesting recent assimilation of C and N likely occurred largely within the boundaries of the habitat type where individuals were captured. Habitat differentiates resource use patterns more strongly than does capture district, which is undoubtedly attributable, in part, to the high degree of habitat heterogeneity in districts. The exception to this pattern is Kohala, which differs from other districts in the abundance of open habitat conditions characterized by pastureland at higher elevations (Maly and Maly 2004) and dry grass‐ and shrubland at lower elevations. This pattern is due to a history of forest clearance and ranching, along with an extraordinarily steep moisture gradient ranging from 150 mm to > 3000 mm mean annual precipitation over a distance of < 15 km (Giambelluca et al. 2013). These conditions make Kohala unusual in supporting a substantially higher proportion of open habitat than other districts (Figure 1) and significant habitat heterogeneity over small spatial scales, which increases the likelihood of differential habitat use and subsistence in open habitat.

**FIGURE 4:**
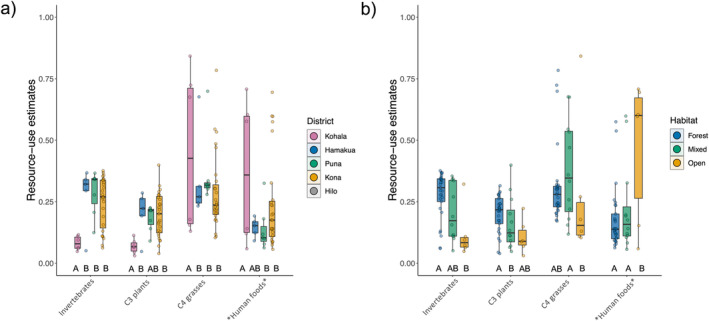
Median resource‐use estimates from Bayesian mixing models separated by (a) district and (b) habitat. Letters signify differences between habitats from SIMPER permutation tests—habitats with no shared letters are significantly different.

Feral pig isotopic and dietary composition followed a distinct pattern along the gradient from forest to open habitat, whereby pigs inhabiting mixed habitats occupied an intermediate position between pigs in forest and open habitats. Our findings suggest that the feral pig dietary niche increases from wetter (forest) to drier (open) habitats as pigs shift toward reliance on resources enriched in both ^13^C and ^15^N (Figure 2). This appears to be due to pigs in forests generally consuming more uniform proportions of each resource category and overlapping in their resource use strategies, while pigs in mixed and open habitats exhibit less uniformity in resource use (Figure 4) and greater variability among individuals (Figures 2 and 3). This leads to divergent patterns characterized by increasing reliance on C4 grasses or animal tissues and human food subsidies as habitats become more open, as evidenced by enrichment in ^13^C and ^15^N, respectively. While several factors may contribute to these patterns, they are likely driven, in part, by differences in resource availability between habitats. Forests, which typically host a higher abundance of available resources—particularly invertebrates and C3 plants—may support a smaller resource niche as individuals capitalize on preferred resources (Lesser et al. 2020). Current commercial agricultural production occurs primarily in wetter regions on Hawai‘i Island and consists largely of C3 crops such as macadamia, coffee, and tropical fruits (Perroy, Melrose, and Cares 2016), which almost certainly play a role in C3 contribution under forested and mixed conditions. Drier sites on Hawai‘i Island, often in the lee of mountains or other orographic barriers (Giambelluca et al. 2013), are typically characterized by a much more dramatic loss of native vegetation historically than forested areas due to past agriculture and livestock grazing, which has largely replaced native vegetation with non‐native C4 pasture grasses. Indeed, a sizeable proportion of individuals in open and mixed habitats exhibited a high proportion of C4 grass in their diet; however, SIMPER revealed that C4 grass proportion was not a significant driver of resource‐use differences between forest and open habitat (Table S4).

^15^N enrichment from forest to open habitat appears to have been driven primarily by lower invertebrate consumption in conjunction with increased reliance on trophically elevated resources in open habitats, such as human food and animal tissues (Table 1; Figure 3). These findings are consistent with patterns we would expect if resource quality—particularly protein content—were a significant driver in shaping diet in these populations. Protein content in forage can act as a metabolic constraint (Barrett 1978; Bowen, Lutz, and Ahlgren 1995; Parker, Barboza, and Gillingham 2009), shaping preferences toward maximizing protein intake given the available resources on the landscape. Protein limitation has been suggested to shape feral pig diet selection in other systems (Baubet, Bonenfant, and Brandt 2004; Belden and Frankenberger 1990; Wilcox and Van Vuren 2009) and has even been offered as a mechanism to explain suspicions that pig population density in Hawaiian forests was low until the introduction of non‐native European earthworms (Loope, Hamann, and Stone 1988). Animal tissues and human foods may satisfy protein requirements for pigs in open habitats in place of invertebrates under forested conditions, supporting this observed trade‐off in open vs. forest habitats (Table 1, Figure 3). Conversely, we did see a small number of individuals in all habitats that relied more heavily on C4 grasses with minimal consumption (< 10%) of other resource categories, which ostensibly contradicts this hypothesis. However, our results indicate N concentrations are higher in C4 grasses than the sampled native C3 plants (Table S1), which, along with supplementation by small quantities of ^15^N enriched protein sources as indicated by mixing models, is possibly sufficient to fulfill metabolic requirements. These requirements seemingly cannot be fully met on a specialized diet of C3 plants, as evidenced by the lack of individuals clustering near the δ‐values for C3 plants in the isotopic mixing space (Figure 2) and estimates of greater invertebrate consumption in forests (Wehr et al. 2020). While the configuration of the mixing space introduces some difficulty in precisely estimating the proportion of invertebrates vs. C3 plants for some individuals, widespread accounts of native plant consumption (Loope, Hamann, and Stone 1988; Nogueira‐Filho, Nogueira, and Fragoso 2009; Wehr et al. 2018) suggest it is highly unlikely that forest pigs clustering near invertebrate N and C δ‐values do not consume C3 plants as a significant portion of their diet. Even in their introduced range, where they feed more heavily on animal material than in their native range (Ballari and Barrios‐García 2014), feral pigs typically prefer vegetative material (Senior et al. 2016).

It is unclear whether reliance on trophically elevated protein sources in place of invertebrates is driven by the scarcity of invertebrates in open habitats (i.e., animal tissue and/or human food as a fallback) or if alternative protein sources are more available in open habitats relative to forests (i.e., animal tissue and/or human food as a preferred resource). The former is suggested by generally lower aboveground net primary productivity and associated resource availability in drier sites in Hawai‘i, which exceeds the marginal decline in productivity in the wettest sites > 2000 mm year^−1^ (Austin 2002). In other systems, human food waste specifically has been observed to serve as a backup when natural resources are unavailable rather than as a preferred resource (Stillfried et al. 2017). The minimal differences in diet found among districts also support this hypothesis, as we would expect higher consumption of anthropogenic resources in districts with high human population density if anthropogenic food subsidies are preferred (Figure 4). Pigs in open habitats may also exhibit larger home‐range sizes (Diong 1982), which would increase the likelihood of exposure to alternative protein sources. Whatever the mechanism, our findings indicate there exist distinct and divergent foraging strategies for pigs on Hawai‘i Island shaped by habitat type. Moreover, we find evidence to suggest contemporary pig populations in open, drier habitats may be sustained by alternative protein sources from those found in wetter, forested habitats.

### 4.2 Individual Specialization in Resource Use

Isotopic signatures and resource use estimates for individuals remained relatively stable through time, with some variability in within‐individual niche breadth. Resource use among individuals across habitat types was consistent with patterns observed above, except for the individual captured in the forest in Kohala. Due to the high degree of habitat heterogeneity in Kohala, it is possible that this individual—while captured in forest habitat—primarily foraged in open grassland, which would explain the high estimates of C4 grasses in its diet. Collectively, these patterns among individuals corroborate findings that pigs employ distinct patterns of resource use and indicate that feral pig feeding strategies, broadly defined by patterns of consumption, do not vary considerably through time. This may be due to the comparatively minor changes in resource availability across time in Hawai‘i relative to regions with greater seasonal variation. Diong (1982) likewise found that pig home‐range sizes in Hawai‘i, which were expected to shift with seasonal changes in resource availability, varied little seasonally in both wet and dry forest habitats. However, seasonal shifts in their distribution and abundance have since been found (Risch, Honarvar, and Price 2022), and other systems at similar latitudes have detected patterns of resource use linked to seasonality, particularly in drier habitats (Wilcox and Van Vuren 2009; Wurster et al. 2012). It also may be that temporal differences in consumption are characterized by variation within, not among, resource categories (e.g., shifting among different sources of C3 plants). Alternatively, pig diets may be relatively stable through time as a result of learned behavior, whereby patterns of resource use remain consistent over time even as conditions shift (Rogers and Blundell 1991; Rozin 1976). On Hawai‘i Island, significant spatial variation occurs over short distances, so individuals may also compensate for changing conditions by moving to access resources. Regardless of the mechanisms, feral pigs in Hawai‘i appear to establish a broadly consistent resource‐use strategy and deviate little through time.

**FIGURE 5:**
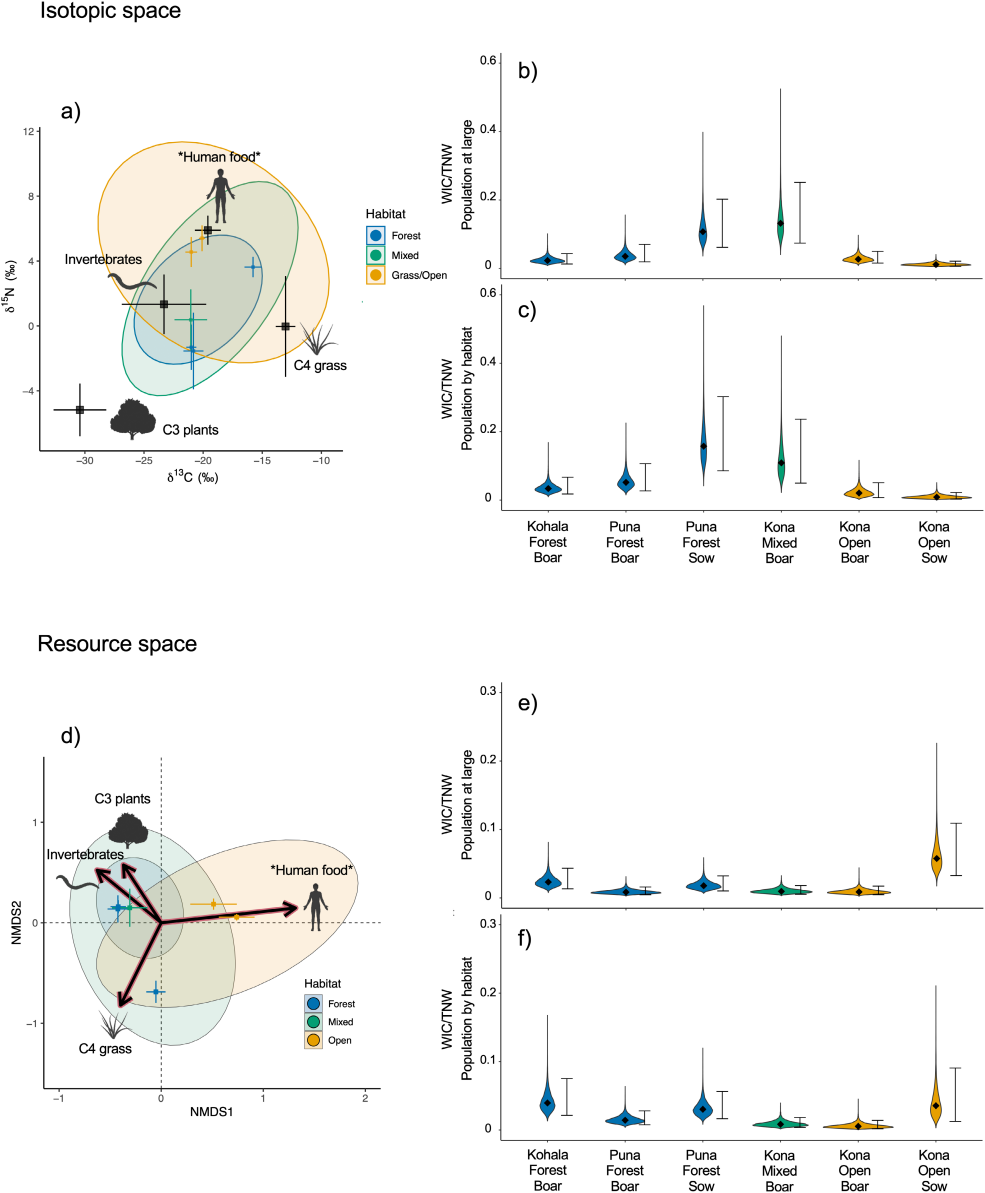
Individual niche width for the 6 chronologically subsampled individuals colored by capture habitat. Error bars show 95% confidence intervals in (a) isotopic space and (d) resource space for each individual. WIC/TNW is given in plots (b) (in isotopic space) and (e) (in resource space), with TWN defined as the niche width for the population at large (i.e., all sampled individuals). WIC/TNW is given in plots (c) (in isotopic space) and (f) (in resource space), with TWN defined as the niche width of the population associated with the habitat where that individual was captured. Violin plots show the posterior distributions for Bayesian SEAc ratios (SEAc for individual/SEAc for population), and error bars represent 95% confidence intervals.

This narrow resource‐use strategy hypothesis is further supported by our finding that individual niche width was relatively small compared to that of the TNW for feral pigs in Hawai‘i. Raw isotopic values and mixing model estimates both demonstrated that while pigs as a species occupy a wide dietary niche, there is substantial intraspecific diet specialization (sensu Bolnick). While our study is limited to a small number of subsampled individuals, every individual sampled occupied only a small portion of available niche space compared to both the overall feral pig niche (Figure 5) and that of pigs found in different districts and habitats (Table S5). Low within‐individual niche width is indicative of conditions under which strong intraspecific competition can drive individuals to specialize in a narrow range of resources or resource proportions (Araújo, Bolnick, and Layman 2011). This may occur either in resource‐poor environments or under high population densities (Kobler et al. 2009; Svanbäck and Persson 2004). Feral pig population density in Hawai‘i is likely high in most areas as they lack any top‐down control aside from human hunting, which is below the levels required to control population growth (Hess and Jacobi 2014). Intraspecific competition is thought to increase in the absence of interspecific competition (Grant and Price 1981; Van Valen 1965), as is likely for pigs in many of the areas where they are found. While feral pigs do overlap with other invasive ungulates (i.e., goats, sheep) in some, primarily drier, sites, they are often the sole invasive ungulate where they establish—particularly in wetter forested areas (Ikagawa 2013; Stone and Anderson 1988). Our findings suggest a high degree of inter‐individual variation and considerable diet specialization in individuals across habitat types, likely driven by high population densities. This may result in variable ecological impacts across individuals and populations, possibly influencing the nature of their effects on communities and ecosystems.

We acknowledge some limitations within our dataset and approach. First, we reiterate that our data come from a non‐random sample of individuals captured by hunters and are unlikely to be fully representative of the population at large. In particular, the full range of demographic and body‐size parameters is unlikely to be adequately represented due to the preferential take of larger individuals. Second, while stable isotope mixing models are powerful tools for inferring dietary composition, they should be interpreted with caution (see: Shipley and Matich 2020). In some cases, results may diverge from other methods such as scat or stomach content analysis. Importantly, isotopic signatures incorporate signals from abiotic processes as well as from diet, which may lead to erroneous interpretations if not carefully accounted for. Additionally, while our isotopic end members represent the primary resources known to constitute the feral pig diet in Hawai‘i, some individuals may rely on other resources not included in our mixing models. Combining more traditional approaches—or others such as DNA metabarcoding—with stable isotope analysis to provide informative priors offers a method of overcoming some of the limitations of our approach. These approaches will become more available as we continue to partner with local hunters. Our results demonstrate the utility of using stable isotope analysis in collaboration with local hunting communities to investigate the trophic ecology of one of the most problematic invasive omnivores in Hawai‘i.

### 4.3 Conclusions

The feral pig dietary niche on Hawai‘i Island is wide, though intraspecific variation and resource availability have led to several distinct feeding strategies. Differences in resource use were broadly shaped by habitat conditions along a gradient from forest to open habitat, with pigs found in forests occupying a narrower resource niche while pigs in open habitats exhibited greater variation, resulting in a broader niche. Dietary choice is likely constrained by protein content, which may explain trophic elevation in pigs as they switch from invertebrate consumption in forests to human food and animal tissues in open habitats. Furthermore, we found considerable individual diet specialization among subsampled individuals, consistent with strong intraspecific competition driven by high population densities. Despite the challenges associated with reconstructing wild animal diets, we were able to identify important drivers of variation in resource use among feral pigs in Hawai‘i by partnering with local hunting organizations.

## Author Contributions

**Michael S. Peyton:** conceptualization (lead), formal analysis (lead), investigation (lead), methodology (lead), resources (equal), visualization (lead), writing – original draft (lead), writing – review and editing (lead). **Kealohanuiopuna M. Kinney:** conceptualization (equal), investigation (supporting), resources (equal), visualization (supporting), writing – review and editing (supporting). **Sarah Knox:** investigation (supporting), resources (equal). **Grace Tredennick:** investigation (supporting), resources (supporting). **Sara Hotchkiss:** conceptualization (supporting), supervision (equal), writing – original draft (supporting), writing – review and editing (equal).

## Conflicts of Interest

The authors declare no conflicts of interest.

## Supporting information


Tables S1–S5

## Data Availability

All data are publicly available on Dryad: https://doi.org/10.5061/dryad.w6m905qx5.
