# Supplementary material for: Dietary Niche Variation in an Invasive Omnivore: The Effects of Habitat on Feral Pig Resource Use in Hawai‘i
Source: Ecol Evol. 2024 Oct 16;14(10):e70417. doi: 10.1002/ece3.70417 (PMC11480647; doi:10.1002/ece3.70417)
Supplement: Supplementary file 1 — Tables S1–S5 [file ECE3-14-e70417-s001.docx]

**Table S1**: ẟ^13^C and ẟ^15^N values (top) and C and N concentrations (bottom) for resource endmembers used in Bayesian stable isotope mixing models

|  | **ẟ13C** |  |  | **ẟ15N** |  |  | **n** |
| --- | --- | --- | --- | --- | --- | --- | --- |
| **Resource** | **mean** | **sd** |  | **mean** | **sd** |  |  |
| Invertebrates | -23.31 | 3.57 |  | 1.33 | 1.84 |  | 7 |
| C3 (native) | -30.42 | 2.23 |  | -5.18 | 1.62 |  | 20 |
| C4 (non-native) | -13.03 | 0.83 |  | -0.037 | 3.11 |  | 12 |
| Human foods | -19.16 | 1.10 |  | 5.90 | 0.90 |  | 1000 |
|  |  |  |  |  |  |  |  |
|  |  |  |  |  |  |  |  |
|  | **C concentration (%)** | |  | **N concentration (%)** | |  | **n** |
| **Resource** | **mean** | **sd** |  | **mean** | **sd** |  |  |
| Invertebrates | 41.31 | 10.19 |  | 8.93 | 3.21 |  | 7 |
| C3 (native) | 47.18 | 3.45 |  | 0.94 | 0.49 |  | 20 |
| C4 (non-native) | 46.08 | 1.36 |  | 1.59 | 0.83 |  | 12 |
| Human foods | 52.80 | 0.45 |  | 6.90 | 0.06 |  | 1000 |

**Table S2**: Results of MANOVA testing differences in ẟ^13^C and ẟ^15^N values in resource endmembers across districts (top) and across different tissue types (bottom). Districts sampled include Hilo and Kohala. Tissues for C3 plants include leaves, roots, and fruits, while tissues for C4 plants include leaves and roots. Asterisk marking invertebrates signifies the lower sample size (n = 7).

|  | **Pillai's trace** | **F** | **df (resid)** | **Pr(>F)** |
| --- | --- | --- | --- | --- |
| **Districts** |  |  |  |  |
| *Invertebrates | 0.50 | 2 | 5 | *0.25 |
| C3 plants | 0.05 | 0.48 | 18 | 0.63 |
| C4 plants | 0.13 | 0.68 | 10 | 0.53 |
|  |  |  |  |  |
| **Tissues** |  |  |  |  |
| C3 plants | 0.09 | 0.39 | 17 | 0.82 |
| C4 plants | 0.13 | 0.69 | 10 | 0.53 |

**Table S3**: Dietary niche space quantified by SEAc in isotopic space (left) and resource space (right) for populations defined by capture district (top) and habitat (bottom). Median values and standard deviations given for each ellipse.

| **SEAc** | **Isotopic space** | | **Resource space** | |
| --- | --- | --- | --- | --- |
| District | median | sd | median | sd |
| Kohala | 11.2 | 6.19 | 0.47 | 0.26 |
| Hāmākua | 12.0 | 7.97 | 0.11 | 0.07 |
| Puna | 7.6 | 3.74 | 0.17 | 0.08 |
| Kona | 15.1 | 3.00 | 0.37 | 0.08 |
|  |  |  |  |  |
| Habitat | median | sd | median | sd |
| Forest | 9.7 | 1.88 | 0.25 | 0.05 |
| Mixed | 17.1 | 5.67 | 0.48 | 0.16 |
| Open | 18.7 | 10.5 | 0.69 | 0.38 |

**Table S4**: Results of SIMPER showing contribution to dietary differences among populations characterized by district (top) and habitat (bottom). Bolded district and habitat combinations on the left-hand column are shown by PERMANOVA to be significantly different at α < 0.10. Bolded values with asterisk are shown by permutation tests to be significant at α < 0.05.

| **SIMPER** | **Invertebrates** | **C3 plants** | **C4 grass** | **Human foods** |
| --- | --- | --- | --- | --- |
| District |  |  |  |  |
| **Kohala vs Kona** | ***0.087** | ***0.069** | ***0.157** | ***0.141** |
| **Kohala vs Puna** | ***0.105** | 0.060 | ***0.153** | ***0.144** |
| Kohala vs Hāmākua | ***0.106** | ***0.074** | ***0.153** | 0.138 |
| Kona vs Puna | 0.055 | 0.043 | 0.084 | 0.084 |
| Kona vs Hāmākua | 0.062 | 0.050 | 0.086 | 0.075 |
| Puna vs Hāmākua | 0.053 | 0.051 | 0.087 | 0.080 |
|  |  |  |  |  |
| **Habitat** |  |  |  |  |
| **Forest vs Open** | ***0.093** | 0.059 | 0.117 | ***0.181** |
| Mixed vs Open | 0.069 | 0.051 | ***0.141** | ***0.172** |
| Forest vs Mixed | 0.067 | ***0.057** | 0.104 | 0.078 |

**Table S5**: Median WIC/TWN for each individual in both isotopic space (left) and resource space (right). Values given with TWN defined as (i) total feral pig niche space, (ii) population niche space defined by capture habitat, and (iii) population niche space defined by capture district.

|  | **In isotopic space** | |  |  | **In resource space** | |  |
| --- | --- | --- | --- | --- | --- | --- | --- |
|  | Total | Habitat | District |  | Total | Habitat | District |
| Kohala.Forest.Boar | 0.02 | 0.03 | 0.03 |  | 0.02 | 0.04 | 0.02 |
| Puna.Forest.Boar | 0.04 | 0.05 | 0.07 |  | 0.01 | 0.01 | 0.02 |
| Puna.Forest.Sow | 0.11 | 0.16 | 0.20 |  | 0.02 | 0.01 | 0.04 |
| Kona.Mixed.Boar | 0.13 | 0.11 | 0.13 |  | 0.01 | 0.01 | 0.01 |
| Kona.Open.Boar | 0.03 | 0.02 | 0.03 |  | 0.01 | 0.01 | 0.01 |
| Kona.Open.Sow | 0.01 | 0.01 | 0.01 |  | 0.06 | 0.04 | 0.07 |
